# Skin immunity in wound healing and cancer

**DOI:** 10.3389/fimmu.2023.1060258

**Published:** 2023-06-16

**Authors:** Arnolda Jakovija, Tatyana Chtanova

**Affiliations:** ^1^Immunity Theme, Garvan Institute of Medical Research, Sydney, Australia; ^2^St. Vincent’s School of Medicine, Faculty of Medicine, University of New South Wales, Sydney, Australia; ^3^School of Biotechnology and Biomolecular Sciences, Faculty of Science, University of New South Wales, Sydney, Australia

**Keywords:** skin wound healing, skin immunity, innate response, skin cancer immunity, skin adaptive immunity

## Abstract

The skin is the body’s largest organ. It serves as a barrier to pathogen entry and the first site of immune defense. In the event of a skin injury, a cascade of events including inflammation, new tissue formation and tissue remodeling contributes to wound repair. Skin-resident and recruited immune cells work together with non-immune cells to clear invading pathogens and debris, and guide the regeneration of damaged host tissues. Disruption to the wound repair process can lead to chronic inflammation and non-healing wounds. This, in turn, can promote skin tumorigenesis. Tumors appropriate the wound healing response as a way of enhancing their survival and growth. Here we review the role of resident and skin-infiltrating immune cells in wound repair and discuss their functions in regulating both inflammation and development of skin cancers.

## 1 Overview

The skin is not only a physical barrier protecting us from infection but also an important immunological site, which in humans contains an estimated 20 billion T cells as well as a range of cells with innate and innate-like roles. Among them are Langerhans cells, dermal dendritic cells (DCs), macrophages, neutrophils, mast cells and innate lymphoid cells. These immune cells, together with keratinocytes and neurons, interact with the skin microbiota, to maintain skin homeostasis while protecting against pathogen invasion. Several recent reviews (1–3) explain how immune subsets and specialized immunological sites (such as hair follicles and sweat glands) interact with the skin microbiome. This review will focus specifically on how skin immune cells mediate wound repair and how this process can be co-opted by tumors.

## 2 Cutaneous tissue injury and wound repair cascade

**Figure 1:**
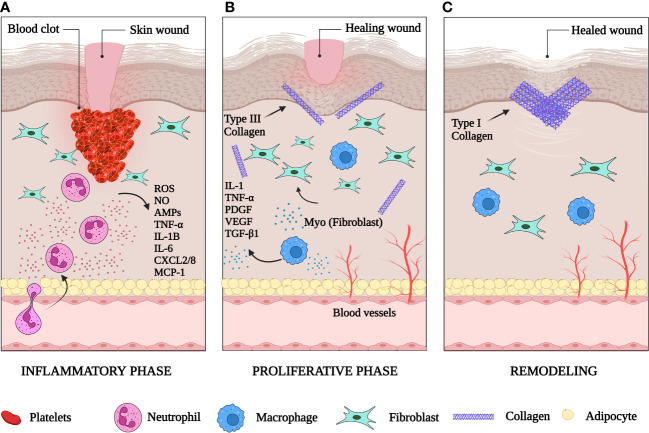
The phases of skin wound healing. **(A)** The inflammatory phase: one to three days after injury the wound is filled with a clot. Inflammatory cells have been recruited to the wound site. Neutrophils release reactive oxygen species (ROS), nitric oxide (NO), antimicrobial proteins (AMPs), TNFα, IL-1B, IL-6, CXCL2/8 and monocyte attracting protein-1 (MCP-1). **(B)** The proliferative phase: macrophages are recruited to clear dead tissue and debris. They secrete IL-1, TNFα, PDGF, VEGF and TGF-β1. New blood vessels form in the wound bed. Fibroblasts are activated in the wound and begin to deposit collagen. **(C)** The remodeling phase: wound contraction occurs, collagen III is replaced by collagen I, and the extracellular matrix is remodeled by proteases and other enzymes. Created with BioRender.com.

Wound healing is a natural physiological reaction to tissue injury designed to prevent the onset of infection and restore tissue integrity (4). It follows a finely coordinated multistep process that includes hemostasis, inflammation, proliferation (new tissue formation), and tissue remodeling (5) (**Figure 1**).

### 2.1 One to three days after injury

#### 2.1.1 Hemostasis and humoral inflammation

Vascular damage with resultant local hemorrhage is a universal characteristic of tissue injury (6). A few minutes after injury, platelets in the circulation begin to stick to the injured site and promote formation of blood clots (7), made up predominantly of crosslinked fibrin, plasma fibronectin and other extracellular matrix (ECM) proteins, such as vitronectin and thrombospondins (8).

#### 2.1.2 Cellular inflammation

Inflammatory cells enter damaged tissues through diapedesis by way of venules within minutes after injury (9, 10). Neutrophils are the first immune subset to respond to cutaneous damage (11). They deploy their antimicrobial arsenal to phagocytose and kill contaminating microorganisms and secrete an array of cytokines that recruit macrophages, T cells and additional neutrophils (12).

Mast cells are abundant in the skin and orchestrate the early stages of wound healing (13). They recognize interleukin (IL)-33 released by necrotic cells via ST2 receptor and secrete histamine and other cytokines that stimulate the immune response (13, 14). This is critical for attracting other immune cells to the wound and promoting inflammation (15).

Monocytes and macrophages follow neutrophils in wounds to remove dead cells and cellular debris and recruit T cells and natural killer (NK) cells to stimulate the proinflammatory response (16). Removal of dead neutrophils by macrophages heralds the end of the inflammatory period and the transition of macrophages to an M2 (anti-inflammatory) phenotype (17) [section 3.2]. This conversion from an M1 pro-inflammatory to M2 anti-inflammatory phenotype is a crucial step in the initiation of the proliferative and resolution phase (18). At the end of this stage, these macrophages either die at the wound or migrate to draining lymph nodes. These events promote subsequent wound healing phases (16) [section 2.2].

### 2.2 One to ten days after injury

#### 2.2.1 New tissue formation

This stage includes angiogenesis, fibroplasia, and re-epithelialization which stimulate the closure of the lesion. Angiogenesis (formation of new microvasculature) enables transport of fluid, oxygen, nutrients, and immune-competent cells into the stroma (19). Fibroplasia commences with the formation of granulation tissue (20) and is characterized by the proliferation of fibroblasts, which deposit the collagen matrix required for adhesion and migration (21). Myofibroblasts, specialized fibroblasts with contractile properties, are responsible for the production of the ECM components that replace the temporary matrix in the wound within the granulation tissue (22). These cells have contractile abilities due to the presence of α-smooth muscle actin (α-SMA) in their microfilament bundles, making them a significant contributor to the contraction and maturation of the granulation tissue (23). The transition from the inflammatory to the proliferative phase occurs two to four weeks after injury as epithelial cells start the process of re-epithelialization that involves their proliferation and migration from the borders of the wound.

### 2.3 One to two weeks after injury

#### 2.3.1 Tissue remodeling

This phase marks the transition from granulation tissue to scar. It starts one to two weeks after wounding and continues for up to two years (24). At this stage, wound tissue is mainly dominated by collagen type I, which has replaced collagen type III (19). This results in the formation of a scar that contains dense connective tissue of reduced tensile strength and elasticity compared with normal skin (25). Granulation tissue is replaced by acellular scar after the completion of wound repair and myofibroblast apoptosis (26).

## 3 The role of immune cells in skin wound healing and cancer

Acute wound healing is a highly regulated process that leads to the restoration of tissue integrity and resolution of inflammation. However, chronic wounds (like diabetic ulcers) can develop if the inflammatory process is not succeeded by the repair phase (27, 28). Many inflammatory skin conditions (such as atopic dermatitis, psoriasis, discoid lupus erythematosus) involve disruptions in immune function and signaling (29). This can result in persistent activation and increased production of pro-inflammatory molecules such as chemokines and cytokines, which exacerbate inflammation and cause abnormal cell growth (30). Diseases such as rheumatoid arthritis and psoriasis also show characteristics of aberrant wound healing (31, 32). Notably, chronic wound state is a risk factor for cancer development (33) and can promote malignant transformation (29, 34).

**Figure 2:**
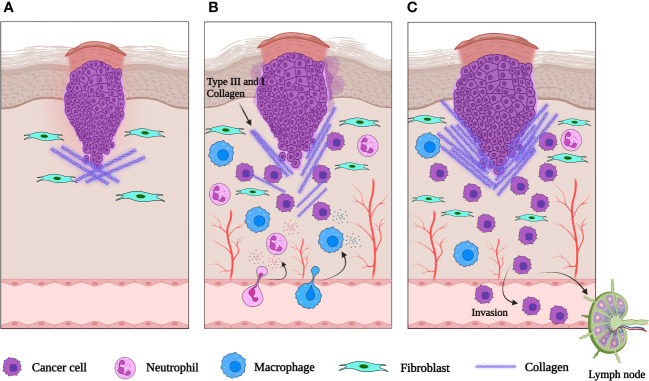
Schematic representation of an epithelial tumor. **(A)** When neoplasia is first initiated, fibroblasts are recruited to the tumor site and activated. **(B)** As the tumor grows, inflammatory cells are recruited to the tumor and release cytokines. VEGF and other signaling molecules induce neovascularization. **(C)** The abnormal extracellular matrix is pro-tumorigenic, pro-angiogenic and increases the invasiveness of the tumor. Created with BioRender.com.

The granulation tissue of healing skin wounds contains a mixture of cells, including fibroblasts, blood vessels, and inflammatory cells. This strongly resembles the tumor stroma suggesting that epithelial tumors promote the formation of their stroma by activating the wound healing response of the host, which leads to the formation of new blood vessels and fibroblasts. This suggests that tumors hijack the proliferative program of wound repair to support their proliferation. The tumor microenvironment (TME) also shapes immune cell function to enhance an immunosuppressive and pro-angiogenic state, aiding tumor immune evasion and promoting metastasis (**Figure 2**). Unlike a wound, the tumor continues to grow uncontrollably, without the resolution of inflammation and proper tissue repair. This evidence has led to the suggestion that tumors represent ‘wounds that never heal’ (35). This idea is supported by the fact that many of the same signaling pathways and cellular players involved in wound healing are also activated in tumor development (36). But while wound healing involves the migration and proliferation of healthy cells to repair the damaged tissue, tumor cells acquire genetic changes that allow them to invade the surrounding tissues and metastasize (36).

In both wound healing and cancer, the initial inflammatory response is necessary to recruit immune cells to the site of injury or to the TME (36). But in chronic wounds or cancer, the inflammatory response becomes dysregulated and promotes further tissue damage, leading to impaired healing or tumor progression (37). Moreover, several studies have demonstrated an association between chronic wounds and skin cancer (38). The specific functions of immune cells can vary depending on the type of cancer and the stage of the disease, and more research is needed to fully understand the role of immune cells in skin repair and skin cancer. Next, we will review in detail the functions of immune cell subsets and inflammatory mediators in cutaneous wound healing and cancers.

### 3.1 Neutrophils

Several molecules attract neutrophils to wounded skin: damage-associated molecular patterns (DAMPs); proinflammatory cytokines, including TNF-α; chemoattractants, such as CXCL1–3 and CXCL8(IL-8); anaphylatoxins C3a and C5a and macrophage inflammatory protein-1α (39, 40). In addition, damaged mitochondria from necrotic cells release other early signals, such as fMet-Leu-Phe (fMLP), derived either from translocated commensal organisms or from necrotic host cell mitochondria (41). At the injury site, neutrophils destroy pathogens via phagocytosis and degranulation, release of highly concentrated reactive oxygen species (ROS), antimicrobial proteins (AMPs) and neutrophil extracellular traps (NETs) (42). They amplify inflammation by secreting cytokines and chemokines, such as TNF-α, IL-1β, IL-6, CXCL8 and CXCL2 (43). Neutrophils also recruit macrophages and T cells via monocyte attracting protein-1 (MCP-1) (44) and play an important role in modulating adaptive immunity in response to infectious wounds (45–47).

LTB4 released from early recruited neutrophils acts as a chemoattractant and mediates an effect known as “neutrophil swarming” (48, 49), a dynamic response to inflammation first observed in neutrophils using two-photon microscopy. Intravital imaging has provided important insight into neutrophil function in skin infection and injury (47, 50, 51). For instance, Lammerman et al. used it to show that the lipid leukotriene B4 was a critical mediator of intercellular signaling among swarming neutrophils after cutaneous thermal injury (48). As neutrophils rearranged the collagen fiber network to create a collagen-free zone at the center of the wound, their clusters were maintained via integrin receptors (48). Real time observation of neutrophil dynamics in zebrafish demonstrated that neutrophil migration to the wound was due to the production of hydrogen peroxide (52).

Neutrophils are not only essential for eradicating pathogens and inhibiting their propagation when the skin barrier is compromised, but also play a beneficial role in the restoration of epithelial tissues. After sterilizing the wound, neutrophils initiate an apoptotic cell-death pathway which leads to efferocytosis by macrophages (53). However, if this process is impaired, neutrophils persist in the wound microenvironment and their associated inflammatory mediators contribute to the formation of chronic wounds (54). Once activated, neutrophils then initiate wound closure, re-epithelialization and formation of new vessels by expressing cytokines and growth factors, including TNF-α and VEGF (40, 55, 56). Neutrophil-derived VEGF plays an important role, for example, in neovascularization of injured murine cornea (57), highlighting neutrophil contributions to restoring tissue architecture.

The importance of neutrophils in tissue repair has been demonstrated in several studies. For instance, mice lacking fMLP receptors 1 and 2 show delays in neutrophil accumulation during the acute stage of injury, resulting in delayed wound closure (58). Likewise, mice deficient in CXCR2, a chemokine receptor important for neutrophil recruitment to the wound site, exhibit delayed re-epithelialization of skin wounds and delayed wound healing (59). In addition, neutropenic patients and mice deficient in the neutrophil protease matrix metalloproteinase 8 (MMP-8) display reduced skin wound repair (60, 61). Interestingly, a new role for neutrophils in wound repair has recently been demonstrated in an internal injury model where neutrophils were shown to carry pre-existing matrix into wounds, promoting fibroblast activation and scar formation (62). Whether such a mechanism also exists in skin injuries is of considerable interest.

In cancer, the release of DAMPS caused by hypoxia, nutrient starvation, cellular proliferation, and necrosis in the TME can recruit and activate neutrophils (63). Tumor and stromal cells can also secrete CXCR2 ligands, such as CXCL1, CXCL2 and CXCL5 to attract neutrophils (64, 65). Within tumors, neutrophils can be located in either the peripheral region or within the tumor core (66). Neutrophils infiltrating the tumor core are less motile compared to the peritumoral neutrophils. The reduction in motility may allow neutrophils to accumulate and promote inflammation (66).

Neutrophil anti-microbial and wound repair functions can be coopted by tumors to mediate immunosuppression and metastasis (67). Neutrophil-derived ROS can suppress T and NK cell responses in tumors (68–70) and activate cellular proliferation or survival signaling pathways, such as the NF-κB pathway and the synthesis of transcription factors like STAT3 (71), which are constitutively activated in skin cancer (72). Oxidative stress regulates the expression of intercellular adhesion protein-1 (ICAM-1), which together with IL-8, controls the transendothelial migration of neutrophils and may contribute to tumor metastasis (73). Consistent with an important role for neutrophils in metastasis, intravital imaging showed that neutrophils are among the first immune cells to arrive at metastatic tumor sites (74), where neutrophil derived NETs act as an adhesion substrate for cancer cells and degrade the extracellular matrix (75–77). Other neutrophil-derived factors, such as granules containing neutrophil elastase (NE), neutrophil collagenase (or MMP8), and gelatinase B (or MMP9) can remodel the ECM in the TME or act directly on tumor cells themselves to boost tumor proliferation and invasion (78). Consistent with this, MMP9 stimulates keratinocyte proliferation and invasion in skin cancer models (79, 80). Tumor neutrophils can release cytokines like oncostatin M, which induces VEGF and increases angiogenesis and tumor cell invasion (81).

Notably, neutrophil-derived mediators of wound repair and pathogen control can also act to eradicate cancerous cells and restrict metastatic dissemination (82, 83). For instance, neutrophils can mediate direct tumor killing by releasing ROS and cytotoxic enzymes, or by recruiting and activating other immune cells, such as cytotoxic T cells (84, 85). This points to a complex role for neutrophils in tumor immunity, where wound repair and pathogen killing mechanisms are applied within the TME in a context and co-stimulation dependent manner which is not yet fully understood.

### 3.2 Macrophages

Skin has two distinct macrophage populations: *tissue resident*, which derive from the extraembryonic yolk sack, and *monocyte-derived*, which originate from the bone marrow-derived monocytes recruited to the skin (86). Tissue resident macrophages monitor the skin microenvironment for signals that indicate cell stress, tissue injury or infection (87, 88). After acute injury they recognize DAMPs and release hydrogen peroxide (89), recruiting neutrophils and monocytes from the blood to further amplify the inflammatory response (90). Tissue resident macrophages are particularly important for the immediate response to injury, while the long-term response is dependent on the bone marrow-derived monocytes which differentiate into macrophages *in situ* (54).

At least three functional subsets of macrophages contribute to the different stages of wound healing and tissue repair: (i) pro‐inflammatory (traditionally referred to as “M1”), (ii) tissue repair or pro-wound healing, and (iii) anti‐inflammatory or pro-resolving macrophages (91, 92). Subsets (ii) and (iii) are collectively referred to as “M2” macrophages. Pro-inflammatory macrophages infiltrate the injury site shortly after the wound is formed to phagocytose and kill bacteria, remove cell debris, toxic metabolites and dead cells (93). They produce inflammatory mediators, such as nitric oxide, ROS, IL-1, IL-6 and TNF-α and secrete MMP-2 and MMP-9 to break down the ECM (94, 95). Macrophage-derived cytokines IL-12/23 and IFN-γ recruit T cells and natural killer cells to amplify the proinflammatory response (16). Pro-wound healing macrophages then release elevated levels of PDGF, insulin-like growth factor 1 (IGF-1), VEGF and TGF-β1 to promote cellular growth and proliferation (96). The function of pro-resolving macrophages is to restore homeostasis, minimize fibrosis via apoptosis of myofibroblasts, and to suppress further T cell proliferation (94). In acute wounds, these macrophages are responsible for tissue repair and neovascularization (97, 98). They also suppress the inflammatory response via secretion of IL-10, arginase 1, resistin-like molecule-α (RELMα) programmed death ligand 2 (PDL2) and TGF-β1, while promoting collagen reorganization and maturation (96, 99, 100). However, macrophages activated through RELMα can also orchestrate pro-fibrotic collagen crosslinking, which is essential for the formation of granulation tissue and progression to a persistent scar (101).

Macrophages are also prominent in the TME, where tumor cells can exploit the macrophage wound repair response (102). In cancer, M1 macrophages inhibit tumor growth, while the M2 phenotype (also known as tumor‐associated macrophages or TAMs) promotes tumor progression (94). TAMs can contribute to different stages of carcinogenesis: initiation, growth, invasion, and metastasis through production of cytokines, growth factors, pro-angiogenic factors, and MMPs (103, 104). For example, the presence of TAMs correlates with increased invasion, micro-vessel density, and COX-2 expression, which are characteristic of more aggressive cancers (105). In squamous cell carcinoma, TAMs have both pro-tumor and anti-tumor activities and appear to be responsible for VEGF-C-induced lymphangiogenesis (106, 107). The macrophage chemoattractant CCL2 is expressed on melanoma cells and regulates macrophage function in melanoma in a concentration-dependent manner (108). Like neutrophils, macrophages can also play a role in preventing skin cancer as intermittent deletion of macrophages can lead to the development of basal cell carcinomas (109).

### 3.3 Langerhans cells and DCs

Langerhans Cells (LCs) are epidermal immune cells of embryonic origin responsible for antigen presentation and the maintenance of tolerance in the skin (110). In severe injuries, which lead to LC loss in the epidermis, cytokines such as MCP1 can facilitate the recruitment of monocytes from the bone marrow which can then differentiate into LCs in the skin (111, 112). In response to trauma, LCs extend their dendrites through epidermal tight junctions and engulf foreign antigens via dendrite tips (113). The presence of antigens can trigger LC activation and migration to the lymph nodes (114), where they can shape T cell responses (115). A subset of skin LCs has been shown to induce the proliferation of resident memory T cells with a regulatory phenotype and their ability to suppress autologous skin resident Tem cell responses (116). This study suggests that the interaction between epidermal LCs and skin resident memory T regulatory cells is important for tolerance to self-antigens and memory response (116).

Dermal DCs are composed of conventional and non-conventional (plasmacytoid) DCs that differ in ontology and functions (117). Conventional DCs are derived from myeloid progenitor cells and are responsible for presenting antigens to T cells, while plasmacytoid DCs which are derived from lymphoid progenitor cells, produce type I interferons (IFNs) in response to viral infections (117). Following skin injury, dermal DCs rapidly migrate toward the site of the injury and surround it (118). Once close to the wound site, these cells can capture cutaneous antigens and deliver them via lymphatic vessels to naive T cells in the draining lymph nodes (119).

The precise contribution of LCs and DCs to skin wound healing is still under investigation. A recent study showed that depletion of langerin+ cells (LCs and a small sub-population of dermal DCs) led to faster wound closure in mice (120). The accelerated wound repair was due to enhanced keratinocyte proliferation in the epidermis and granulation tissue formation, suggesting that langerin+ cells inhibit keratinocyte proliferation during wound healing (120). On the other hand, in another study, loss of CD11c+ cells (LCs and DCs) resulted in failure of wound closure (121). In particular, re-epithelization did not occur, and the wounds remained completely open. Since depletion of langerin+ cells removes LCs, as well as a small sub-population of langerin-expressing dermal DCs, while leaving the majority of dermal DCs unaffected, these studies suggest that dermal DCs may have a pro-reparative role, whereas LCs may hinder tissue repair. This is supported by a study showing that LCs can produce TNF which can contribute to tissue damage (122). It is worth noting that mice lacking TNF exhibit improved wound healing (123). These studies point to the important roles of LCs and DCs in wound healing, but the precise contribution of each subset may depend on the type of injury and other cells in the microenvironment.

LCs and dermal DCs are often the first immune cells to encounter antigens from cutaneous cancers (124). The effectiveness of the immune response against tumors may depend on the ability of LCs and DCs to present antigens and activate anti-tumor T cells (125). For example, in squamous cell carcinoma (SCC), there is a reduction in the number of LCs and dermal DCs, which can disrupt the generation of adaptive immunity. In SCC, DCs are poor stimulators of T cell proliferation compared to their peritumoral or healthy skin counterparts (125). In contrast, LCs harvested from SCC lesions have been found to have an increased ability to stimulate CD4+ and CD8+ T cells *in vitro*, compared to LCs from healthy skin (126). LCs are potent stimulators of T cell responses making them optimal targets for immunization strategies against melanoma (127), especially since the spontaneous regression of melanoma in humans is often linked to a T cell predominant infiltrate into the lesion (128). LCs may also play a role in the epithelial–mesenchymal transition (EMT) in cutaneous cancers, due to the involvement of molecules that regulate LC migration and EMT (129).

### 3.4 Lymphocytes

Chemokines produced in the wound including CCL3, CCL4 and CCL5 (130) attract conventional T cells to the wound site. Recruited T cells can be found in murine wounds within 24 hours of injury and persist for 30 days (131). This long timeframe suggests that they may have important roles not only during inflammation but during the proliferative and remodeling phases. For example, cytotoxic T cells release substances that kill microorganisms and clear the infection (20). T cells can also participate in the later stages of wound healing, where they exert several functions: clearance of damaged cells and debris, regulation of immune response and prevention of excessive inflammation, promotion of angiogenesis and ECM remodeling (131).

Lymphocytes differentiate into various subsets to create specialized immune responses, such as helper T cells (Th1, Th2, Th17), innate lymphoid cells (ILC1, ILC2, ILC3), and unconventional T cells (γδ T cells, iNKT cells, MAIT cells) (132). These responses can be classified by the cytokines they produce e.g., IFN−γ for type 1 immunity, IL-4, IL-5, and IL-13 for type 2, and IL-17 and IL-22 for type 3 (133). Type 2 responses play an important role in maintaining homeostasis and repairing tissue damage, and are coordinated by tissue-resident cells like ILC2s, which expand after injury (134). For instance, healthy skin of naïve C57BL/6 mice contains a population of resident ILC2s that expand after wounding (135). The importance of ILC2s has been demonstrated in mice lacking IL-33 (which contributes to the expansion of ILC2s in both humans and mice) (136). Impaired re-epithelialization in these mice is associated with diminished numbers of activated ILC2s at the site of injury (135).

In addition, mouse epidermis is enriched for γδ T cells and CD8^+^ resident memory T (T_RM_) cells. CD8 T_RM_ cells are sessile non-circulating cells and can appear after the resolution of skin inflammation caused by infection (137). γδ T cells have a T cell receptor (TCR) composed of γ and δ subunits and demonstrate characteristics normally associated with both innate and adaptive lymphoid cells. They are abundant in mouse but not in human epidermis and play a major role in wound healing. For instance, the lack of skin γδ T cells is associated with decreased inflammation and delayed wound resolution (138).

T cells are also a crucial component of the immune system’s response to cancer. They recognize cancer cell antigens to generate an anti-tumor immune response and can control certain infections and cancers including those located in the skin. The presence of CD8^+^ T cells in melanomas as well as in other cancers is associated with better clinical outcomes (139, 140). However, the TME can impair CD8+ T cell ability to respond to tumor antigens as a result of activation of checkpoint proteins, such as PD-1 and CTLA-4 (141, 142). The combination of immune checkpoint inhibitors, specifically anti-CTLA-4 and anti-PD-1 antibodies, is now providing an effective therapeutic strategy in many cancers, including advanced melanoma, for which tumor regression and long-term durable cancer control is possible in nearly 50% of patients (143, 144). Multiple studies have demonstrated that NK cells can also exert significant anti-tumor effects (145, 146). In particular, they have been shown to recognize and destroy melanoma cells *in vitro* and *in vivo* (147).

The regulatory T cell subset (Tregs) plays a balancing role in inflammation by suppressing the underlying immune response. However, increased number of Tregs in sites of chronic skin inflammation did not resolve the injury, but actively delayed wound healing (148). In tumors, e.g., melanoma, Treg infiltration is a poor prognostic indicator (149, 150). Intravital analysis of Treg behavior *in vivo* revealed that Tregs in the TEM are migratory, in contrast to the surrounding CD8 T cells, and form unstable contacts with CD11c+ APCs. This leads to a reduction in the levels of costimulatory molecules and the activation of inhibitory receptors, such as PD-1 and TIM-3, on CD8+ T cells (151).

## 4 Concluding remarks

Impaired responses to injury result in the development of chronic wounds, which have a major impact on the quality of life (152, 153). Yet there are few treatments available once the processes leading to non-healing chronic wounds, aberrant scarring and fibrosis have begun. This makes regulation of inflammatory pathways, and especially the switches between acute and chronic inflammation, attractive targets for intervention with treatments that could be relevant to non-healing wounds. One potential new approach to achieve resolution of inflammation in non-healing wounds or cancer is to target specific inflammatory pathways (18, 30). There are a number of therapies under investigation, such as immunomodulatory agents, which may reduce inflammation and promote healing (154). For instance, animal studies have shown that cytokines, such as IL-10, which dampen inflammation, can enhance wound healing (155, 156). Furthermore, a recent study demonstrated the efficacy of IL-10 in reducing inflammation, accelerating wound healing and reducing scarring in two preclinical murine models (157). In the same study, a phase II randomized controlled trial demonstrated the translation of this therapeutic effect from animals into humans (157). Stem cells or regenerative therapies can also be used to promote tissue repair (158, 159). Several clinical trials have utilized various types of adult stem cells to improve wound healing (160, 161). Although none of these treatments have been officially approved as of yet due to major limitations such as stem cell immunogenicity and their reduced survival *in vivo* (162), this research highlights how understanding of the mechanisms of wound repair can lead to the development of novel therapies for large or non-healing wounds.

Since the cellular and molecular players involved in generating wound stroma can be co-opted in cancer to build tumor stroma, understanding the mechanisms of stroma generation in wounds may suggest approaches that prevent tumor stroma generation. For example, the use of anti-angiogenic therapies, which target the formation of new blood vessels, has proven successful in cancer treatment, with anti-VEGFA antibodies currently being used to treat patients with metastatic colorectal cancer (163, 164). Likewise, analysis of how inflammation is subdued once the wound is repaired may aid the development of immunotherapeutic strategies for cancer treatment.

The growth and spread of cancer cells depend on the establishment of a microenvironment, which shares a lot of commonalities with the wound healing processes. Nuanced understanding of the immune system’s role in wound repair over the whole process, including not only angiogenesis and immunosuppression, but also its potential contributions to rebuilding structural integrity of the wound and re-establishing immune networks, is essential for the development of better approaches for promoting wound healing, and the advancement of novel antitumour therapies.

## Author contributions

TC conceived and developed the manuscript. TC and AJ contributed to writing and editing this manuscript. All authors contributed to the article and approved the submitted version.
